# Bridging the Knowledge Gap for the Impact of Non-Thermal Processing on Proteins and Amino Acids

**DOI:** 10.3390/foods8070262

**Published:** 2019-07-17

**Authors:** Sara Esteghlal, Hadi Hashemi Gahruie, Mehrdad Niakousari, Francisco J. Barba, Alaa El-Din Bekhit, Kumar Mallikarjunan, Shahin Roohinejad

**Affiliations:** 1 Department of Food Science and Technology, School of Agriculture, Shiraz University, Shiraz, Iran; 2 Biomolecular Engineering Laboratory, Department of Food Science and Technology, School of Agriculture, Shiraz University, Shiraz, Iran; 3 Nutrition and Food Science Area, Preventive Medicine and Public Health, Food Science, Toxicology and Forensic Medicine Department, Faculty of Pharmacy, Universitat de València, Avda.Vicent Andrés Estellés, s/n 46100 Burjassot, València, Spain; 4 Department of Food Science, University of Otago, Dunedin 9054, New Zealand; 5 Department of Food Science and Nutrition, University of Minnesota, St. Paul, MN 55108, USA; 6 Burn and Wound Healing Research Center, Division of Food and Nutrition, Shiraz University of Medical Sciences, Shiraz, Iran

**Keywords:** Irradiation, ultrasound, cold plasma, pulsed electric fields, high-pressure processing, proteins and amino acids

## Abstract

Proteins represent one of the major food components that contribute to a wide range of biophysical functions and dictate the nutritional, sensorial, and shelf-life of food products. Different non-thermal processing technologies (e.g., irradiation, ultrasound, cold plasma, pulsed electric field, and high-pressure treatments) can affect the structure of proteins, and thus their solubility as well as their functional properties. The exposure of hydrophobic groups, unfolding followed by aggregation at high non-thermal treatment intensities, and the formation of new bonds have been reported to promote the modification of structural and functional properties of proteins. Several studies reported the reduction of allergenicity of some proteins after the application of non-thermal treatments. The composition and concentration of free amino acids could be changed after non-thermal processing, depending on the processing time and intensity. The present review discusses the effects of different non-thermal treatments on protein properties in detail, and highlights the opportunities and disadvantages of these technologies in relation to protein functionality.

## 1. Introduction

Food proteins may undergo several chemical modifications during their processing [1]. Numerous physical, chemical and microbiological changes may occur due to the diversity of food-processed products, and the knowledge of their detailed mechanisms still needs greater research efforts to maximize the quality and stability during production and storage. The main difficulty in elucidating these mechanisms is related to the complexity of the changes occurring and the limitations of current analytical methods that are faced dealing with intractable food matrices most of the time. Technological processes that are used for food preservation and production may affect the functional, nutritional, and biological properties of food proteins. While some changes may impart health-related benefits, such as the generation of biologically active peptides [2], others may affect amino acids and generate toxic derivatives, such as lysinoalanine, D-amino acids, and biogenic amines [3]. Several non-thermal technologies have been proposed in order to reduce the impact of conventional thermal processes on food matrices such as Maillard reactions, changes in color, flavor, physicochemical composition, and so on. Ultrasound, UV irradiation, cold plasma, high-pressure processing, and pulsed electric fields are among the most investigated/applied non-thermal food processing technologies, and some of them are already have been industrialized [4,5]. Knowledge regarding their impact on proteins and amino acids is scarce and not well explained in the literature, despite the numerous benefits of these emerging technologies over conventional food processes, such as time and energy saving, reducing solvent quantity, which matches the “green” processing concept [6]. This review aims to fill this knowledge gap by examining the impact on non-thermal processing methods on structure and functionality of proteins.

## 2. Proteins and Amino Acids

Proteins are macro biopolymers that play an important role in the cells of living organisms. The structure and components of the animal and vegetal cells (e.g., lipoproteins, enzymes, hormones, antibodies, globulins, etc.) have a protein base that plays a critical role in the viability and functioning of these cells. Animal and plant proteins provide 35% and 65% of the world’s protein requirement based on the report of Young and Pellet [7], respectively.

Amino acids are the structural units of proteins and peptides [8] and they contain carbon, hydrogen, nitrogen, and oxygen as the main atoms of their structure. Among all of the known amino acids, only 20 (standard amino acids) are proteinogenic, meaning that they have a triple codon in the DNA. There are two non-standard amino acids, selenocysteine, which is abundant in eukaryotes and non-eukaryotes, and pyrrolysine, which is found in bacteria and archea [9]. In comparison to carbohydrates and lipids, proteins are rich in nitrogen (≈15–25%). Each amino acid contains a carboxyl group (-COOH), an amine group (-NH_3_), and a side chain (R group) bonded to a core carbon atom. The side chain of each amino acid may contain other atoms, including sulfur and phosphorus. Amino acids could be categorized into alpha (α), beta (β), gamma (γ), and delta (δ) based on the location of the functional groups on the core carbon. Polarity (polar and non-polar), pH (acidic and alkaline), and side chain (aliphatic, acyclic, aromatic, containing hydroxyl or sulfur, etc.) can also be used as classification tools.

Amino acids (both proteinogenic and non-proteinogenic) play an important role in biosynthesis and neurotransmitter transport. For instance, γ-amino butyric acid (GABA, non-standard amino acid) and glutamate (standard glutamic acid) are among the most important inhibitory and excitatory neurotransmitters in the human brain, respectively. On the other hand, glycine (standard amino acid) is necessary in the formation of porphyrins, which are used in red blood cells, whereas proline is used to synthesize hydroxyproline, which is the main component in collagen [10].

Four different structures of proteins have been identified, including primary, secondary, tertiary, and quaternary structures. In the primary structure, two amino acids are bound to each other by a peptide bond, which is an amide bond between the carboxylic acid group (-COOH) of one amino acid and an amine group (-NH_2_) of another amino acid. When the number of bonded amino acids in a chain is less than 50, it is called peptide, and when the number of residues is more than 50, it is called polypeptide or protein. The secondary structure of proteins is based on inter-strand or intra-strand hydrogen binding. The two main kinds of secondary structure are the α-helix and β-sheet. The former is a right-handed coiled strand in which hydrogen bonds are formed between the oxygen of C=O group of each peptide bond and the hydrogen bond of N-H group of another peptide bond that was located in the fourth amino acid below in the same chain. In the β-sheet structure, hydrogen bonds are formed between two different strands, which can be paralleled or anti-paralleled. The tertiary structure is the three-dimensional shape of a polypeptide/protein. Hydrogen, hydrophobic, di-sulfide bonds, and/or electrostatic interactions between the side chains of amino acids induce protein folding to reach the maximum stability. Hydrophobic groups are located inside the protein molecules, while the hydrophilic ones are exposed outside. The interaction (e.g., di-sulfide and salt bridges, hydrophobic interactions, and hydrogen bonds) between some polypeptide chains forms their quaternary structure as a complex aggregate [10].

Amino acid composition, sequence, and structure (primary, secondary, tertiary, and quaternary) determine the molecular weight, net charge, physicochemical, and functional properties of proteins. Some of these properties that are directly related to food quality include hydrophobicity, solubility, thermal stability, gel forming, emulsifying ability, swelling, water holding capacity (WHC), and association/dissociation behaviors that dictate aspects, such as color, flavor, and texture of foods [10].

Human cells cannot synthesize essential amino acids (phenylalanine, valine, threonine, tryptophan, methionine, leucine, isoleucine, lysine, and histidine), and thus humans rely on diet to obtain them. Animal and vegetal sources of proteins have different nutritional values and protein qualities. Animal proteins include all of the essential amino acids and they have large similarity with human ones. However, good sources of high animal proteins, such as meat, have high cholesterol and fat contents, as well as high sodium content in some cases, which can limit the consumption of meat [11]. Overall, vegetal proteins have lower nutritional quality than the animal ones due to the limitation of the essential amino acids, with the exception of some, which have been found to be suitable substitutes for meat, also that there is a difference in the protein digestibility between the animal and vegetal proteins.

## 3. Food Processing

### 3.1. Conventional Thermal Processing

Food processing is usually a required set of steps applied to food before its consumption. The main reasons for food processing include: imparting a desirable modification in the food composition, maintaining the food quality, sustain the availability of products at various times and places (products provision in out of season), food diversity (creating diverse food products to different consumers), increasing shelf life, and preparation of ready-to-eat products [12,13]. Changes occurring during processing can be beneficial, such as the inactivation of microorganisms and destruction of toxins, increasing the bioavailability of some nutrients, development of desirable flavor and texture attributes, and extending shelf life, or it may be detrimental, such as the destructive effect of heat on nutritional value of food (e.g., loss of vitamins and bioactive compounds) and the formation of harmful components (e.g., acrylamide, trans fatty acids) [14], Moreover, a loss of amino acids can occur, depending on the severity of the treatment.

**Figure 1:**
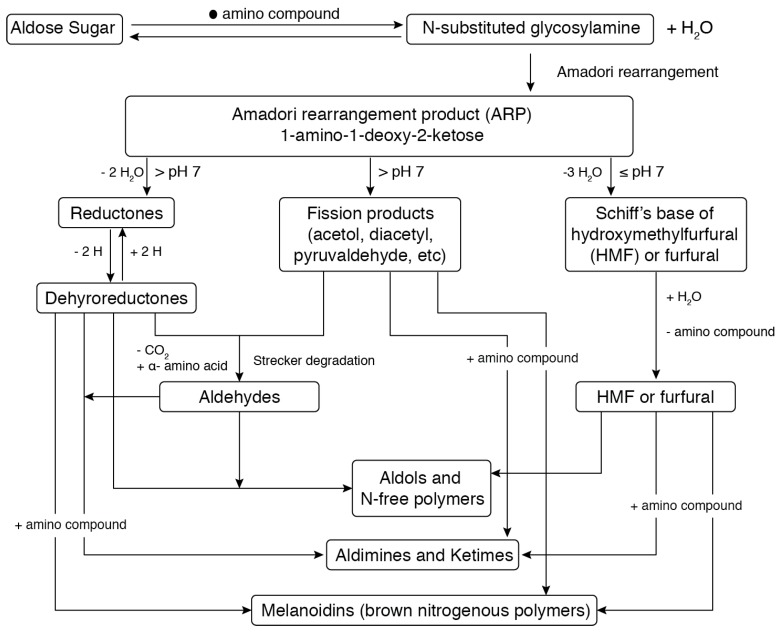
Scheme of Maillard reaction. Adapted from Hodge [21].

The most usual traditional processes in food industries include heating, cooking, baking, freezing, milling, canning, fermentation, drying, salting (pickling, curing, or brining), extrusion, and smoking [15]. Among these processes, thermal processing (e.g., cooking, roasting, grilling, frying, boiling, pasteurizing, and sterilizing) are considered to be the most efficient in destroying pathogens, but they also have the most drastic effect food on composition, characteristics, and properties [16]. For example, proteins could be denatured during heating, depending on the temperature and the protein in question, causing the loss of their quartnary and tertiary structures and forming unfolded random shapes. Additionally, thermal treatment (90 °C, 2 h) of proteins gives rise to the formation of isopeptides, lysinoalanine, and racemization [17], alter proteins’ allergenicity and stretching of some amino acids along with their peptide bonds in the primary structure [18]. Proteins during heat processing are stimulated to interact with other components in the food system. Maillard reactions are one of the most important, which involve proteins and contribute greatly to the nutritional and sensory properties of foods. The reactions are initiated by interactions between reducing sugars and amino acids and they continue with a large set of chain reactions. These reactions may affect the color, flavor, and aroma of the food product, cause the formation of toxic compounds (e.g., acrylamide, furans, and hydroxyl propyl furfural), and decreased the digestibility and nutritional value [19,20]. Figure 1 shows a scheme of the Maillard reaction [21].

### 3.2. Emerging Non-Thermal Processing

Although thermal processing can contribute and assure the safety of foods, it can reduce the nutritional and sensory properties [6,22]. When considering the consumers’ demand for high quality (high nutritional value, fresh taste, and desirable sensory properties, such as color and texture), strategies for minimally processed foods and alternative non-thermal technologies to conventional processing have been developed to produce microbiologically safe, fresh, and nutritious foods. These innovative food processing technologies include mainly ultrasound (US), UV irradiation, cold plasma (CP), high pressure processing (HPP), and pulsed electric fields (PEFs) [23,24]. Low energy and water requirements, as well as a higher efficiency and environmentally friendly nature, make these non-thermal techniques more preferred than the traditional thermal ones [25]. The effect of the non-thermal processing technologies on protein structures, functionalities, and allergenicity will be described in this review.

## 4. Impact of Non-Thermal Processing on Proteins

### 4.1. Surface Hydrophobicity

Table 1 shows a summary of the effects of non-thermal properties on proteins and amino acids. The number of hydrophobic groups on the surface of proteins determines their hydrophobicity [26]. Some functional properties of proteins (emulsifying, foaming and gel-forming), as well as their stability and conformation, are dependent on the hydrophobic interactions.

Non-thermal treatments can affect the surface hydrophobicity of proteins by imposing conformational changes, such as unfolding (partial or full unfolding, depending on the processing conditions) and displacement of hydrophilic and hydrophobic groups [27].

The sensitivity of a protein toward a denaturation treatment (thermal or non-thermal) also depends greatly on its structure. The stability of tertiary and/or secondary protein structures in denaturation treatment is related to hydrogen interactions between the polar groups and interactions of non-polar groups (hydrophobic interactions) through the surrounding water molecules, which form cages around hydrophobic groups. Electrostatic bonds and Van der Waals interactions are also involved in the denaturation process, although to a lesser extent [28]. For example, α-lactoglobulin is more resistant to high pressure treatment (and thermal treatments) when compared to β-lactoglobulin due to its four intramolecular disulfide bonds as compared to the two for β-lactoglobulin and its linked calcium ion that also help to maintain its stability toward denaturation. In another example, β-sheet structures are more resistant to pulsed electric fields as compared to α-helical structure. β-sheet structures, whose peptide chains are arranged in a pleated way and are also stabilized by hydrogen bonds, are not as widespread. In comparison to in α-helical structures, the side groups of β-sheet inherent amino acids are very closely arranged to one another, such that similarly or bulky charged residues disturb this structure type. Consequently, larger areas of β-sheet structure may only be formed in the presence of small residues, as can be found in alanine or glycine, whereas larger residues lead to polypeptide chain α-helical formations [29].

For instance, Kang [26] evaluated the effect of ultrasound power on beef proteins during the brining process. Based on their results, longer treatment time and intensity involve increased surface hydrophobicity. They have demonstrated that higher treatment time and/or ultrasound intensity causes the exposure of interior hydrophobic groups and as a result of an increase in the surface hydrophobicity. These findings were the same to those that were reported by other authors for pork proteins that were treated by ultrasound and H_2_O_2_ [30]. Zhang et al. [31] evaluated the effect of ultrasound on myofibrillar proteins (MP) and found that the cavitation phenomenon in ultrasound treatment denature the hydrophobic amino acids from the interior parts to the surface, and thus increases the surface hydrophobicity. In their study, a positive correlation was reported between the surface hydrophobicity of the proteins and their solubility. It should be noted that the correlation between these two factors (protein surface hydrophobicity and protein solubility) depends on the strength of the intramolecular interactions within a protein and the involvement of exposed hydrophobic groups in these interactions. For example, more participation of exposed hydrophobic amino acids in intramolecular interaction results in less protein solubility [32]. The presence of electrostatic interactions may explain some of these contradictory results. For instance, stronger electrostatic interactions (as compared to intramolecular ones) can prevent the aggregation by keeping proteins at a far distance from each other, and thus increasing the protein solubility.

Zhang et al. [31] also reported that the number of thiol (-SH) groups was decreased in broiler proteins, as affected by high intensity ultrasound, which is probably due to the formation of intermolecular disulfide bonds (S-S). Hydrogen peroxide, which was produced from water molecules during ultrasound treatment, can oxidize thiol groups to disulfide bonds [26]. The authors also suggested that the available thiol groups that were located inside the proteins could be exposed to the surface and this phenomenon could explain the equal amount of free thiol groups and the reactive forms after treatment by 100 W of ultrasound waves. The evaluation of the pulsed ultrasound on squid mantle proteins showed an increase of 108.39% and 243.08% in the surface hydrophobicity while using continuous and pulsed treatments, respectively. The highest content of hydrophobic amino acids was reported at the tail of the α-helix structure of myosin [49]. Similarly, the application of pulsed ultrasound on bovine serum albumin (20 W.cm^−1^, 45 min) [50] and egg white proteins (20 kHz, 15–30 min) [51] showed an increase by 24% and 35% in surface hydrophobicity, respectively. These observations are related to the protein unfolding, which makes the reorientation of hydrophobic regions possible. Protein characteristics and concentration, aqueous medium, energy and time duration of pulsed ultrasound can influence the proteins unfolding and as a result, the exposure of hydrophobic amino acids that were located inside the proteins to the surface. The effect of pulsed ultrasound on duck liver protein isolate (UDLPI) was also investigated [36]. The authors used a fluorescent probe (8-anilino-1-naphthalene sulfonic acid; ANS) with weak fluorescence in an aqueous medium. The intensity of the ANS fluorescence was greatly increased after ultrasound treatment (24 kHz, 266 W, on-time 2 s and off-time 3 s for 42 min), and it indicated a 39.5% increase in the surface hydrophobicity due to the exposure of aromatic residues to the aqueous medium. The protein surface net charge depends on the nature and the amount of the available charged amino acids. The increase in zeta potential value of the UDLPI (related to the surface net charge) confirmed the irreversible conformational changes in protein structure and the exposure of charged amino acids to the solvent. It has been previously reported that ultrasound treatment causes the formation of free radicals, and it is the main reason for the increased net charge [52].

Segat et al. [44] investigated the effect of atmospheric pressure cold plasma (ACP) on solution containing whey protein isolate (WPI) while using the fluorescent probe “ASN” to detect the conformational changes on proteins. The authors suggested that the enhanced fluorescence intensity after long duration (30–60 min) of ACP treatment was due to the molecular unfolding and exposure of hydrophobic amino acids from the inside part of protein structure to the surface.

### 4.2. Structural Changes and Aggregation

**Figure 2:**
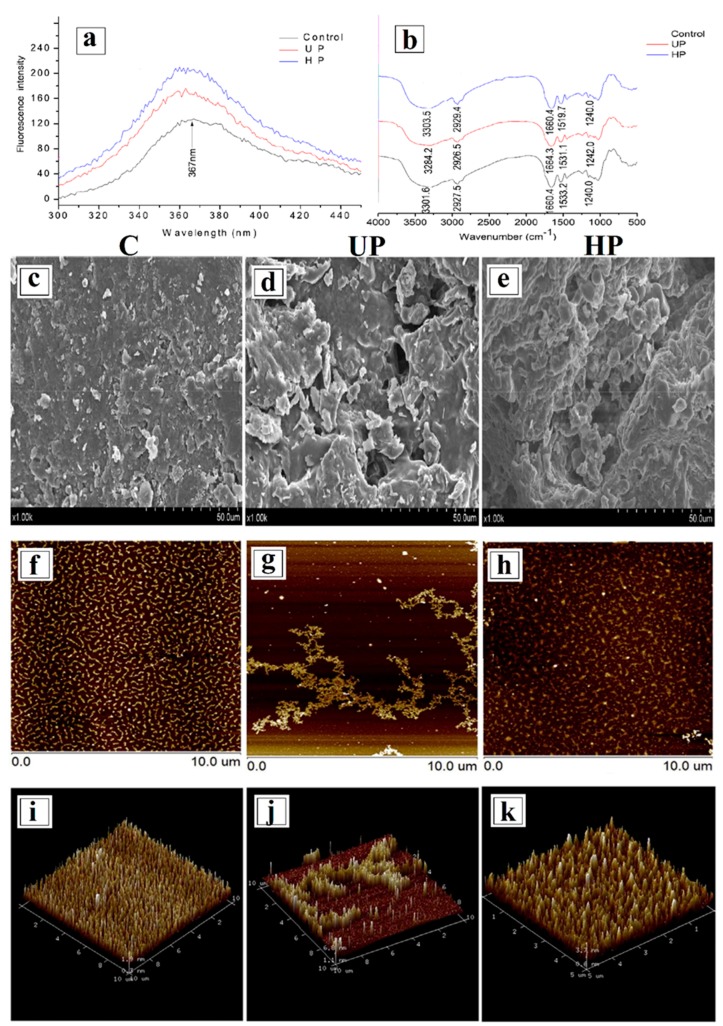
Effect of heat and ultrasound pretreatments on the fluorescence spectra (**a**), Fourier Transform Infrared (FTIR) spectra (**b**), scanning electron microscopy (SEM) (**c**–**e**), atomic force microscopy (AFM) microstructure (**f**–**h**), and three-dimensional (3D) images (**i**–**k**) of corn gluten meal. C, Control; UP, Ultrasound pretreatment and HP, Heat treatment, respectively. Reproduced with permission from [33].

Changes in the primary structure of proteins could be detected by evaluating the variations of the amount and type of amino acids that were present. The effect of heat and ultrasound pre-treatments on the fluorescence spectra, Fourier Transform Infrared (FTIR) spectra, scanning electron microscopy (SEM), atomic force microscopy (AFM), and three-dimensional (3D) images of corn gluten meal are shown in Figure 2 [33]. The SDS-PAGE analysis is usually used to examine the protein profile and determine protein hydrolysis or protein-protein interactions. Ultrasound treatment at a frequency of 20 kHz for 30, 60, 90, and 120 min was performed on fresh beef [26]. The results showed that when the process was carried out for more than 120 min, higher molecular weight polymers were formed, and the myosin heavy chain was reduced under non-reducing conditions (i.e., in the absence of β-mercaptoethanol) at all tested ultrasound intensities (2.39, 6.23, 11.32, and 20.96 W.cm^−2^). This led the authors to suggest the formation of cross-links by disulfide bonds as the mechanism responsible for myosin aggregation. Disulfide bonds may involve free radicals that formed due to cavitation during ultrasound treatment [26]. Other authors have reported that the covalent and non-covalent bonds were the main interactions that caused the aggregation of muscle proteins that were treated by ultrasounds [32,53]. However, it should be noted that some researchers have reported no effect for ultrasound treatment on protein–protein interaction. For instance, it has been shown that the banding pattern of squid mantle proteins did not change after shorter ultrasound treatment for 30, 60, and 90 s at a frequency of 20 kHz [49].

Similarly, the electrophoresis profile of chicken breast was not affected by ultrasound (20 kHz, 5, 10, 20, and 30 min) [54]. Giving the obvious differences between chicken and squid (white meat) and beef (red meat), it will be interesting to investigate the impact of iron as a catalyst for the protein–protein interaction. Pulsed ultrasound at a frequency of 25.6 kHz for 30 min on giant squid mantle protein caused protein hydrolysis, which was observed by electrophoresis as the reduction in the size of the proteins’ bands. The use of a combination of time and frequency to ensure the absence of changes in the functional properties of proteins is desired during the processing of food while using ultarsound. Pulsed ultrasound (at a frequency of 24 kHz a power of 266 W, at 2 s on and 3 s off for 42 min) did not affect the protein profile (as shown by the bands on SDS-PAGE) of duck liver protein isolate, which means that the primary structure and peptide bonds were resistant to the ultrasound treatment [36]. Pulsed ultrasound treatment for 15 min at 240 W on chicken myofibrillar proteins did not affect the primary structure as the bands for myosin heavy chains. Actin and tropomyosin were similar for the pulsed ultrasound treated and non-treated samples [35]. These results show that the extent of protein susceptibility to hydrolysis is highly dependent on the protein source and structure.

Analytical methods, such as Fourier Transform Infrared (FTIR) and Circular Dichroism (CD) spectroscopies, could be used to detect the alteration of secondary structure, including α-helix, β-sheet, random coil, and β-turn [26]. Based on the results of Kang et al. [26], Jiang et al. [55] and Herrero [56], observed that ultrasound treatment caused protein unfolding, and consequently decreased the α-helix and increased the β-sheet contents in the treated protein samples.

It was suggested that the increase in β-sheet content, which is accompanied by protein aggregation in some cases [56], was partly dependent on the exposure of the hydrophobic groups [30]. Zou et al. [36] used the DICHROWEB procedure to determine the proportion of various secondary structures of duck liver protein isolate. They reported a decrease of 30.5% and 26.2% of α-helix and random coil and an increase of 152.6% and 42.3% of the β-sheet and turn structures after ultrasound treatment, respectively. According to the authors, the destruction of bonds between some of the amino acids and some distinct parts of proteins followed by protein aggregation caused the observed structural changes. In contrast to these results, a decrease in the β-sheet structure and a 38-fold increase in the α-helix structure of whey protein concentrate were reported after ultrasound treatment at a frequency of 20 kHz and power of 250 W. Wang et al. [35] reported that the secondary structure of chicken myofibrillar proteins was composed of 33.31% α-helix and 18.72% random coiled polypeptides. After 3–15 min of pulsed ultrasound exposure (Table 1), the β-turn content was increased, whereas the α-helix and β-sheet contents were significantly decreased. The authors demonstrated that the random coil content was increased until the 6th min and then decreased. They explained that the partial destruction of inter- and intra-molecular hydrogen bonds in β-sheet and α-helix structures, respectively, caused some degree of unfolding and created an unordered structure that could convert into random coils and β-turns.

The sensibility of a protein toward a denaturation treatment will depend on its structure. Changes in the secondary structures (as detected by CD-analysis) of soybean protein isolate (SPI) after PEF treatment (at a constant PEF strength of 30 kV/cm after 0, 144, 288, and 547 μs) and secondary structure of bovine immunoglobulin G at a PEF strength of 41.1 kV/cm after 54 μs were reported to be insignificant [57,58]; Yeom et al. [59] and Yang et al. [60] found that PEF treatment led to a loss in α-helix structure of papain and β-sheet structure of pepsin, as well as the inactivation of these two enzymes.

The presence of colloidal calcium phosphate in casein micelle induce the formation of the quaternary structure, which is sensitive to high-pressure treatments (˃200 MPa) [61,62,63]. At these levels of pressure (˃200 MPa), the HPP of casein micelles induced the calcium phosphate to dissociate from the micelle causing the disruption of the micelles [61,62]. Acid-induced casein gel that was treated by pressure or had a pressure pre-treatment, showed more strength and rigidity and less syneresis by increasing the pressure [64]. The coagulation time of rennet in the pH range of 5.5–7 was considerably reduced after high-pressure treatment at 200 MPa. Casein in the absence of calcium phosphate does not have tertiary structure and hence is not influenced by high pressure and denaturation, aggregation, and coagulation do not occur after high-pressure treatment [63]. After high-pressure treatment, an increased viscosity of bovine milk, a decreased turbidity (due to casein micelle dissociation), and protein sedimentation occurs [61,62,65]. It has been reported that the treatment of milk at pressures higher than 400 MPa lead to a decrease in the optical properties of milk. As the optical properties (turbidity or L value) are influenced by the size and number of casein micelles, the reduction in optical properties is attributed to the dissociation of calcium phosphate, disruption of casein micelles, and consequently smaller micelles. The high pressure-induced reduction in turbidity is reversible on storage at temperatures higher than 10 °C, but not at 5 °C [62].

### 4.3. Particle Size/Molecular Weight Distribution and Zeta Potential

Studies on the effect of ultrasound treatment on myofibrillar proteins (240 W), chicken myofibrillar protein, duck liver protein isolate (24 kHz, 266 W, 2s on, 3 s off for 42 min), and milk showed a decrease in particle size after sonication [31,35,36]. Turbulent flow and micro-streaming, caused by cavitation, agitated protein aggregates vigorously, dissociated them, and reduced the particle sizes. More homogeneous chicken myofibrillar proteins and a narrow distribution of duck liver protein isolate were obtained after ultrasound treatment, which is likely to have significant impact on the texture and mouthfeel properties of the products. After 3 min of ultasound exposure, chicken myofibrillar protein particles with irregular form were changed into a filamentous form. The smallest and more distinct particles as a result of more unfolding and exposure of hydrophobic groups appeared after 6 min The size of the particles at 15th min of treatment was larger than that at the 3^rd^ min This was explained by the more hydrophobic interactions between proteins as the treatment time increased [35]. Gülseren et al. [50] reported an increase in the BSA particle size after sonication, which was the result of the formation of small aggregates.

The particle size and polydispersity index (PDI) of whey protein isolate (WPI) did not change after 15 min of atmospheric cold plasma treatment. The particle size and PDI increased after the 15th min and the 30th min, respectively, up to the end of the treatment (60 min). The unfolding of protein molecules followed by the formation of a network was proposed as the probable reason for these changes [44].

**Figure 3:**
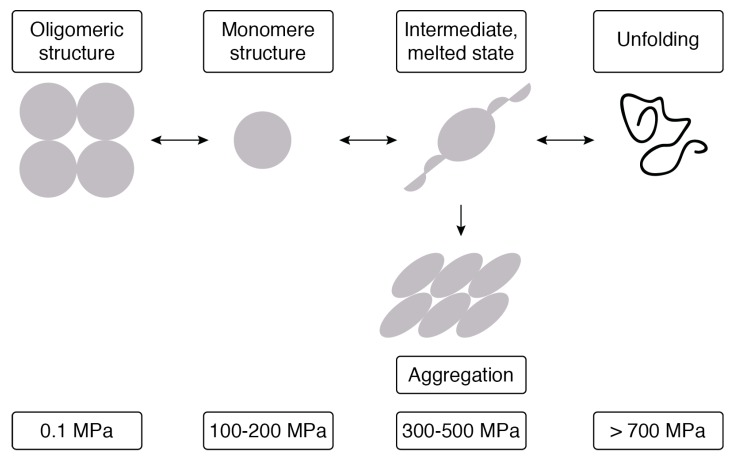
Scheme of protein structure modification by high-pressure processing. Adapted from Bolumar et al. [68].

Li et al. [58] reported that PEF treatment at longer treatment times caused SPI aggregation, as well as an increase in the average particle size and molecular weight. Laser light scattering indicated that non-treated SPI had a wide range of size distribution between 100 nm and 1000 nm, with a mean diameter of 166 nm. A treatment time of 72 µs had no effect on particle size and distribution when compared to the control. However, after 144 µs, the average particle size was 218 nm in a wide range of size distribution. Particles that were bigger than 1000 nm (average 4107 nm with 4.58% intensity) and less than 100 nm (average 28.66 nm diameter with 7.42% and 261.1 nm diameter with 88% intensity) at higher PEF treatment times (above 144 µs and more distinctly after 547 µs) were the result of aggregation and dissociation, respectively. This result implies the different effects of PEF on aggregates, depending on the type of bond maintaining these aggregates (covalent vs. non-covalent). An increase in the particle size due to protein aggregation was also reported [66] after high-pressure treatment at 400 MPa and 600 MPa on soy protein isolate (SPI). These results concurred with the molecular weight distribution that was determined by size-exclusion chromatography. The molecular weight distribution of non-treated SPI was similar to the PEF treated samples for 72 µs and 144 µs, a poly-dispersion distribution with two main peaks of 158 kDa, which were related to 7 s and 11 s, and 67 kDa related to 7 s after dissociation. The peaks of proteins with a molecular weight higher than 1000 kDa were related to 11s and 7s regions. Longer treatment times (288 µs and 547 µs) led to protein denaturation that was followed by aggregation. As a consequence, peaks of 158 kDa and 67 kDa became lower, whereas the peaks of high molecular weight proteins (˃1000 kDa) became more dominant [58]. Concerning the effect of the high-pressure treatment that was applied in the range of 100–1000 MPa on the structure of globular proteins, it has been reported that the formation of hydrophobic and electrostatic interactions is associated with extreme changes in volume, while hydrogen bonds are accompanied by small volume changes (reference). Thus, high-pressure treatment can disrupt the ion pairs and the structures that were formed by hydrophobic interactions, without affecting the hydrogen bonds. Secondary protein structures that are stabilized by hydrogen bonds do not change significantly due to high-pressure. However, tertiary and quaternary structures that mainly include hydrophobic and electrostatic interactions are sensitive to high-pressure and that could be changed to cause protein denaturation, followed by aggregation and gelation. Di-sulfide bond has shown an important role in the formation and structure of high pressure-induced gels [63,67]. The scheme of protein structure modification by high-pressure processing is shown in Figure 3 [68].

### 4.4. Solubility and Gel-Forming/Stability

Ovalbumin shows a relative stability to pressures below 400 MPa and it does not form a gel at this pressure due to the presence of strong non-covalent bonds and four disulfide bonds that stabilize its structure. A pressure-induced gel could be formed at pressures higher than 400 MPa [63]. In biopolymers where hydrogen bonds are dominant, such as gelatin, a higher pressure (≅600 MPa) is required for stable gel [69].

Pressure-induced gels from vegetable proteins can be formed at 300 MPa for 10–30 min treatment [70]. Elastic moduli of these soft gels are significantly lower than the heat-induced ones [71]. Solid behavior of wheat proteins after high-pressure treatment was reported to be due to the weakening effect of pressure on hydrogen bonds and hydrophobic interactions ([71]. Higher pressures resulted in more solid-like and dense gel due to the formation of disulfide bonds.

The interaction between pressure and temperature is an important factor in determining the effect of high-pressure treatment on sarcoplasmic proteins. Marcos et al. [72] reported that increasing pressure that was caused the reduction of solubility and extractability of sarcoplasmic proteins [73,74]. Protein extractability is a direct measure of the quality of sarcoplasmic proteins [75,76]. The denaturation of these proteins and formation of aggregates accompanied by intermolecular disulfide bonds as a result of pressure treatment are the reasons for solubility and extractability reduction. In the range of 200–400 MPa, no effect of temperature was observed, while, at 600 MPa, lower solubility was observed for the samples that were treated at 30 °C as compared to those that were treated at 10 °C [77].

According to Sun & Holley [78] and Cheftel & Culioli [79], pressure causes sarcoplasmic proteins to depolymerize, followed by increased solubility, which facilitates gelation and tenderization. It is worth noting that in this regard, the intensity of pressure is very important, and it will control the outcome of the treatment, especially in fresh meat. The pre-treatment of pressure at 100–300 MPa has been shown to tenderize meat [80,81], however pressures above 300 MPa had an opposite effect [82,83]. Better gelation of meat proteins at pressures below 400 MPa as a result of more solubility of proteins was reported [84], thus leading to more tender meat. A three-fold increase in protein solubility was reported in red meat after high-pressure treatment at 150 MPa at 0 °C for 5 min [85]. Actin was the most sensitive protein in meat and it underwent some degree of modification at 100 MPa [86]. A pressure of 140 MPa was considered as the starting point of changes in the meat proteins. Actin oligomers were formed by head-to-head interactions at that pressure, while the helical structure of the tail was unaffected. Myofibrillar and sarcoplasmic proteins of meat were mostly denatured at 399–400 MPa, and irreversible denaturation occurred above 400 MPa [58].

The use of SDS-PAGE to evaluate the effect of high pressure on meat proteins was reported [87,88]. Differences among the protein bands before and after high-pressure treatment were observed by following the protein band intensities. An increased intensity of myofibrillar proteins was related to increased solubilization, due to depolymerization and interactions between protein ingredients and water, whereas decreased intensity was the result of denaturation, followed by aggregation or insolubilization. Ohshima et al. [87] reported that some parts of fish sarcoplasmic proteins were covalently bonded to each other, and thus could not be extracted or detected by SDS-PAGE. Molina et al. [89] reported that a high-pressure treatment of SPI at 400 MPa decreased the protein solubility due to aggregation, while a pressure of 200 MPa had no effect on the solubility. Li et al. [58] found that the solubility of SPI was proportionally increased with the time and PEF intensity up to a certain value. PEF intensity of 30 kV/cm (at a constant treatment time of 228 µs) showed a maximum solubility of 82%, which was probably due to protein unfolding and subsequent interactions with water. More treatment intensity or time caused the SPI solubility to decrease due to denaturation and aggregation.

High-pressure treatment oxidizes thiol groups of β-lactoglobulin into intermolecular disulfide (S-S) bonds and creates aggregates [90]. This effect was more pronounced at high concentrations. When compared to heat-induced β-lactoglobulin gel, an acid-induced gel was found to be fragile and it has a porous structure with less cross-linking [91]. This gel with its sponge-like structure showed high syneresis and it was sensitive to exudation [92].

### 4.5. Emulsifying Properties

The size of droplets of oil-in-water (*o*/*w*) emulsion stabilized by ovalbumin before and after the pressure pre-treatment of β-lactoglobulin below 600 MPa did not change significantly, however larger droplets were found after applying pressure pre-treatments above 600 MPa [93]. Pressure causes emulsion droplets to flocculate and the more the protein concentration and pressure level, the more flocculation was observed. A similar result was observed about 11s globulin protein of soy, when it was used as an emulsifier [89]. The stabilizing and emulsifying properties of 11s globulin were reduced after pressure pre-treatment due to the formation of intermolecular disulfide bonds that led to aggregation. When compared to the high pressure treatment (800 MPa, 30 min), a mild heating process (80 °C, 5 min) on ovalbumin, and 11s globulin (as emulsifier) caused more flocculation and consequently larger droplet size [63,93]. Emulsions containing pressurized (up to 800 MPa) whey proteins or β-lactoglobulin as emulsifier had bigger droplets than the unpressurized one. Despite the increase in hydrophobicity after high-pressure treatment, structural changes in protein decrease its emulsifying properties, however the gel-like structure of protein that is induced by pressure increases the viscosity of the system and thus stabilizes the emulsion. High-pressure treatment of emulsion stabilized by low β-lactoglobulin content did not affect the size of droplets or creaminess. A higher level of flocculation occurred after pressure treatment in the presence of a high amount of free (non-absorbed) proteins in the emulsion, due to the unfolding, aggregation, and free proteins acting as a bridge between the dispersed droplets by cross-linking them [94].

### 4.6. Reduction in Allergens

A reduction in allergenicity can occur due to the structural changes, including denaturation (loss of the secondary, tertiary, and/or quaternary structures), inter-molecular and intra-molecular (covalent or non-covalent) interactions, breaking disulfide bonds, and aggregation [18].

Meinlschmidt et al. (2016) investigated the effect of remote and direct cold atmospheric pressure plasma (CAPP) on the immunoreactivity of Gly m5 in soy protein isolate (SPI). The electrophoresis pattern of SPI was changed after 2.5 min treatment by remote and direct CAPP at 9 kV voltage. The disappearance of some protein bands in the SDS-PAGE of CAPP treated SPI was attributed to the formation of the insoluble proteins. Plasma offers reactive forms of nitrogen (N_2_, NO, NO_2_), oxygen (O_2_, O_3_, OH), and also UV-A and UV-B, which seriously affect proteins and promote protein-protein interactions or cross-linkage of free amino acids to proteins. As a consequence, protein solubility is simultaneously decreased by the formation of insoluble aggregates. According to the results of sandwich ELISA with mouse monoclonal anti Gly m5 bodies, the direct and remote CAPP treatment of soy protein caused a 100% and 89% reduction in immunoreactivity of Gly m5, respectively.

Direct CAPP at 30 kV and 60 Hz for 5 min at ambient temperature resulted in a 76% reduction in tropomyosin allergen in shrimp [95] and 37% reduction in wheat allergen potency [96]. Based on the study of Tammineedi et al. [97], the antibody binding ability of whey and α-casein were not altered by remote CAPP. Structural and conformational changes in the binding sites of allergens were the reason for the immunoreactivity alteration of allergens. For instance, hydroxyl radical from the plasma treated sample can attack peptide and disulfide bonds and form RSH and RSO radicals. Oxygen radicals can also cleave the peptide bonds. These free radicals can affect the binding sites of sensitive amino acids and change them [29,46].

Pal et al. [45] showed that the banding pattern of grain rice flour did not change after 5 min and 10 min of plasma treatment. Changes in the physicochemical properties of rice after plasma treatment could thus be attributed to starch changes. In contrast to this result, Wan & Yu [98] reported that ozone treatment could oxidize proteins and form disulfide bonds. As consequence, the FTIR spectrum of protein after the treatment showed a strong adsorption at 1042 cm^−1^, which refers to disulfide bonds in cysteic acid.

Gamma-irradiation can cause the loss of conformational epitopes, depending on the dose used. Consequently, this leads to a decrease in the antibody-binding capacity and the allergenicity due to the depolymerization and aggregation of allergen proteins, followed by changes in conformational epitopes [46]. The destruction of an epitope at high doses due to cleavage of disulfide bonds, decarboxylation, and deamination by free radicals may thus occur [99]. It has been also reported that applying gamma-irradiation induces the depolymerization and aggregation of proteins, and the alteration of IgE binding epitopes in milk [48], shrimp [99,100], and egg [101] allergens. Oxidation (by oxygen radicals created during water radiolysis), fragmentation, and inter-molecular bindings of proteins (including hydrophobic interactions and disulfide bonds) are the main chemical changes of the proteins after γ-irradiation [102]. Meinlschmidt et al. [46] reported that the reduction in intensity of Gly m5 and Gly m6 bands after γ-irradiation in SDS-PAGE is dose-dependent. At 5 kGy and 25 kGy, slight fractures in Gly m5 and Gly m6 occurred, but aggregation was not observed. Above 25 kGy, Gly m6 started to decrease and at 100 kGy it was completely destroyed. According to the results of Meinlschmidt et al. [46] and Vaz et al. [103], γ-irradiation at lower doses (3 kGy and 5 kGy) increased the allergenicity of Gly m5 and Gly m6 in SPI and Con-A, respectively. This might have been due to the partial unfolding and aggregation of species at low doses, which can lead to increased exposure of linear and conformational epitopes. Reduced allergenicity was observed for shrimp tropomyosin, egg white ovalbumin, and legume proteins after the exposure at 25 kGy and 100 kGy of γ-irradiation. Lower allergenicity of heat-sensitive shrimp proteins after irradiation was observed in another work [104]. This was due to the cleavage of covalent bonds by photon energy or reactive oxygen. 10 kGy is the maximum dose of γ-irradiation that could be applied to foods to ensure human safety, based on the FDA reports.

The photo-chemical, photo-physical, and photo-thermal effects of pulsed UV (PUV) can alter the binding sites of antibodies as a result of conformational and structural changes, such as cross-linking or aggregation and epitopes alteration [105,106]. Molecular ionization that occurred by free radicals also has a significant effect on epitope changes and reducing the immunoreactivity after PUV treatment [105]. Meinlschmidt et al. (2016) reported that, based on the result of SDS-PAGE, the Gly m5 protein band of cold plasma-treated SPI was less intensive than the untreated sample. The Gly m6 and Gly m5 bands were completely vanished after 6 min and 2 min at the distance of 10 cm and 8 cm, respectively. In this research, sandwich ELISA showed that a reduction in band intensities of PUV-treated SPI was due to protein cross-linking and aggregation that led to an increased protein molecular weight.

## 5. Impact of Non-Thermal Processing on Amino Acids

Several studies evaluated the effect of emerging processing technologies on the stability of amino acids. For instance, Garde-Cerdán et al. [47] reported that the total concentration of amino acids that was found in Parellada grape juice was not affected by PEF treatment; while, the concentrations of phenylalanine, histidine, asparagine, tryptophan, and ornithine were higher in the PEF-treated grape juice when compared to the non-treated samples. PEF treatment probably caused electroporation that disrupted the vacuoles where the proteases are trapped and led the enzymes to release. Smaller protein fragments and free amino acids probably appeared due to the effect of released protease on proteins. Changes in the contents of amino acids were insignificant and initial amino acid profiles were still present in grape juice after PEF treatment [47].

Evaluating the effect of non-thermal plasma (NTP) on total amino acid composition of long and short grain rice flours revealed that aspartic acid, glutamine, and arginine content were decreased after NTP treatment, while serine, glutamic acid, asparagine, histidine, tryptophan, threonine, GABA, isoleucine, proline, and phenylalanine were increased in both long and short grain rice flours. The total content of aromatic amino acids increased after NTP treatment, whereas basic and acidic amino acid contents decreased in both types of flour. The effect of NTP treatment appeared to be amino acid-dependent. For instance, citrulline content was increased in short grain flour and decreased in long grain flour after treatment. Glutamic acid, serine, and glutamate were the most three affected amino acids by NTP, respectively [45].

In a study by Zhou et al. [107], the total content of amino acid was proportionally decreased to the ozone concentration. Changes in the composition of amino acids after ozonation were probably related to their oxidation. The side chain of glutamate and aspartate induced slow reactions with ozone. Amino acids containing sulfhydryl groups are the first amino acids that react with ozone, followed by tryptophan, tyrosine, and histidine, respectively [108].

Segat et al. [44] reported that NPT oxidized cysteine presented in WPI solution and decreased the free thiol groups. The changes in amino acid content were insignificant in Brussel sprout seedlings after high-pressure treatment at 200–800 MPa, and also in brown rice grain and soybean soaked in water after high-pressure treatment at 200 MPa [109,110]. It is likely that high-pressure treatment was a mild treatment that can only affect the non-covalent bonds and small molecules were not affected [111]. High-pressure treatment can change the free amino acid content by affecting the physicochemical reactions by which free amino acid content increases during the growth of seeds (protein hydrolysis or cellular biosynthetic activity). Therefore, cell disruption and inactivation/degradation of individual molecules appear to be the consequences of high-pressure impact on amino acids [43]. In the work that was performed by Barba et al. [43], a treatment time of 3 min was not sufficient for changing the contents of free amino acids. Concerning the effect of high pressure on individual amino acids, alanine, glutamine, glycine, aspartic acid, leucine, phenylalanine, tyrosine, proline, serine, and tryptophan were slightly affected by pressures up to 800 MPa, while asparagine, valine, histidine, glutamine, isoleucine, lysine, and threonine were not affected. Among the amino acids in the affected group, the concentration of glutamine and asparagine were increased after high-pressure treatment, however, the concentrations were decreased for others. These ten free amino acids were affected at various pressure levels, mostly at 200 MPa [43]. Peñas et al. [42,112] reported that high-pressure treatment in the range of 100–300 MPa for 15 min at 37 °C increased the proteolysis of soybean whey proteins (and free amino acids content) due to the increase in proteolytic enzyme activity. This was in agreement with Eisenmenger and Reyes-De-Corcuera [113], who found that high pressure can induce the stabilization and activation of some enzymes. In this regard, the level of pressure that was used in the treatment and also enzyme types are the factors that determine the efficiency [43].

Takai et al. [114] investigated the effect of atmospheric cold plasma on 20 amino acids in aqueous solution while using high resolution mass spectroscopy. The results showed that the amount of aromatic and sulfurous amino acids decreased after the plasma treatment. Fourteen amino acids were converted to new derivatives and products due to their side chains oxidation. Aromatic amino acids, including phenyl alanine, tyrosine, and tryptophan, were hydroxylated and nitrated. Methionine was sulfoxinated and the other thiol containing amino acid, cysteine, was sulfonated and formed the disulfide bond. Proline and histidine underwent ring-opening and amidation during the cold plasma treatment. The oxidation products of lysine, arginine, glutamine, glutamic acid, valine, leucine, and isoleucine were also observed by mass spectroscopy after atmospheric cold plasma treatment. It has been reported that the aromatic and sulphurous amino acids are the most susceptible amino acids to be modified by cold plasma.

As reported by Khoroshilova et al. [115] {Khoroshilova, 1990 #8013}, the disulfide bond in cystine and aromatic amino acids can be photolysis by UV. Based on the result of Surowsky et al. {97}, quenching occurred in tryptophan emission spectrum after cold plasma treatment of peroxide and polyphenol oxide enzymes, which was attributed to tryptophan oxidation and also decreased distance between tryptophan and hem resulting in higher energy transfer between them. Increasing in the emission wavelength after cold plasma treatment, was evidence for the conversion of tryptophan to hydroxyl products (*N*-formylkynurenine and kynurenine) that have emission at around 440 nm.

## 6. Conclusions

The effect of different types of non-thermal treatments, including ultrasound, high pressure, cold plasma, irradiation, and PEF on some of the protein properties was discussed. Protein hydrophobicity can be increased after non-thermal treatments due to the unfolding and exposure of hydrophobic amino acids. Different hydrophobic and disulfide interactions could occur as a result of exposing the thiol groups and hydrophobic amino acids to the surface. Changes in the proportion of different types of secondary structures as a result of non-thermal treatments have been reported in various investigations. Protein solubility and particle size could be altered in different ways (e.g., an increase in size and decrease in solubility due to aggregation/decrease in size, because of turbulent flow/increase in solubility due to more interaction points with water after unfolding) after non-thermal treatments. Hydrogen bonding, and thus the gel-forming ability of proteins can also be changed by non-thermal treatments. The relationship between the reported modifications and sensory aspects by consumers are rarely addressed and future research should address this gap. Some researchers reported the decrease of the allergenicity of some proteins after non-thermal treatments due to changes in the antibody’s binding ability. The content of amino acids and their concentrations has been also reported to change in some studies. The consequence nutritional impact of the changes in amino acids profile will affect the bioavailability, limiting amino acid as well as sensorial aspects as free amino acids could change the flavor of food, and these effects are yet to be determined. Further studies are needed to determine the effect of other non-thermal processing methods, such as pulsed light on the physicochemical properties of proteins and amino acids. Moreover, most of the studies were performed on the laboratory-scale and for a commercial pathway; it is important to evaluate their effects on the industrial scales. Overall, the non-thermal processing technologies are providing the food industry with opportunities to modify food and impart changes beyond the traditional food safety that can be of great importance to consumers and industry.

## Figures and Tables

**Table 1:** Effect of non-thermal processing on proteins and amino acids.

Treatment	Substrate	Condition	Results	Reference
Ultrasound	Corn gluten meal	40 kHz, pulsed on- 10 s and off 3 s, 40 min and 20 °C.	- Molecular unfolding and exposure of hydrophobic groups - Decrease in α-helix and increase in random coil contents after heat/ultrasound and ultrasound/heat treatments	[33]
	Soy protein	20 kHz, power 65 W, 0.5, 1, 5 & 15 min	Protein extraction yield enhanced due to increasing in the solubility	[34]
	Beef proteins	2.39, 6.23, 11.32 and 20.96 Wcm^−2^, 30, 60, 90 and 120 min	- Increase in S0 and decrease in -SH groups - Myosin aggregation and formation of higher molecular weight polymers - Decrease in α-helix and increase in β-sheet contents	[26]
	Myofibrillar proteins	200, 400, 600, 800 and 1000 W, 88, 117, 150, 173 and 193 Wcm^−2^	Increase in S0, decrease in particle size	[31]
	Squid (*Dosidicus gigas*) mantle proteins	20 kHz, 0, 20, and 40%), 0, 30, 60, and 90 s	- Hydrophobicity was increased - The content of reactive sulfhydryl didn’t change - Better emulsifying ability	[27]
	Chicken myofibrillar protein	240 w, 0, 3, 6, 9, 12 and 15 min)	- Increase in -SH groups - No changes in primary structure - Increase in β-turn and decrease in α-helix and β-sheet structures - Decrease in particle size, narrow size distribution	[35]
	Duck liver protein isolate	24 kHz, 266 W by a pulsed on-time of 2 s and off-time of 3 s for 42 min	- Increase in S0 - No changes in -SH content, primary structure and peptide bonds - Decrease in α-helix and random coil and increase in β-sheet and turn structures - Decrease in particle size	[36]
	Β-Lg In Cow Milk	9.5 W, 135 W/cm^2^	No significant alteration in allergenicity	[37]
	Tropomyosin from shrimp	30 Hz, 800 W for 30–180 min	Allergenicity was reduced	[38]
High pressure processing	Hongqu Rice wines	200 and 550 MPa, 25 °C, 30 min	Free amino acids content was decreased after 6 months storage	[39]
	Brown rice	0.1–500 MPa,10 min	Free amino acids especially essential ones were increased	[40]
	Tropomyosin from shrimp	200, 400 and 600 MPa at 20 °C for 20 min	- Conversion of α-helix structure into β-sheet and random coil - Free sulfhydryl content was decreased - Surface hydrophobicity was increased by increasing the pressure from 200–400 MPa and decreased at the range of 400–600 MPa - Allergenicity was decreased	[41]
	Soy allergen (Glycinin)	100, 200 and 300 MPa for 15 min	- Polyelectrolysis was increased	[42]
	Brussels sprouts	200 and 800 MPa for 3 min, 5 °C	- The total free amino acids content was constant - The concentration of glutamin and asparagine were increased	[43]
Cold plasma	Whey protein isolate	70 kV, 1, 5, 10, 15, 30, and 60 min	- Unfolding and exposure of hydrophobic amino acids - Particle size and PDI after 15 and 30 min of treatment were increased - Free -SH groups were decreased - Oxidation of cysteine	[44]
	Grain rice flour	-	- No changes in protein bands were observed - Total aromatic acid concentrations were increased, and acidic and basic amino acid contents were decreased - The most affected amino acids were glutamic acid, serine and glutamate	[45]
Pulsed ultraviolet light	Soy protein isolate (SPI)	1, 2, 4 and 6 min Three pulses per second with a width of 300 μs	Vanishing Gly m5 & Gly m6 bands after few minutes and decrease in allergenicity	[46]
Cold atmospheric pressure plasma		1, 2.5, 5, 7.5 and 10 min without stirring	Reduction in immunoreactivity of SPI	
Gamma-irradiation		Target doses were 3, 5, 10, 25, 50, and 100 kGy	Decrease in SPI allergenicity (Gly m5 & Gly m6) was dependent on the irradiation dose	
Pulsed electric field	Grape juice	4 µs width and with a field strength of 35 kV/cm, 1000 Hz and the total time 1 ms	- Increase in the concentration of phenylalanine, histidine, asparagine, tryptophan and ornithine - The total concentration of amino acids did not change	[47]
Radiation	Β-Lg in cow milk	3, 5, and 10 kGy	Protein aggregation and alteration of IgE binding epitopes	[48]

Gly: Glycinin, SPI: Soy protein isolate, kGy: Kilo gray
